# Retraction Note: Polymerase Spiral Reaction (PSR): A novel isothermal nucleic acid amplification method

**DOI:** 10.1038/s41598-023-43187-7

**Published:** 2023-09-25

**Authors:** Wei Liu, Derong Dong, Zhan Yang, Dayang Zou, Zeliang Chen, Jing Yuan, Liuyu Huang

**Affiliations:** Institute of Disease Control and Prevention, Academy of Military Medical Sciences, Beijing, 100071 China

Retraction of: *Scientific Reports* 10.1038/srep12723, published online 29 July 2015

The Authors have retracted this Article, because the amplification mechanism of the reported method is not supported by the data presented.

After publication of this Article concerns were raised that the proposed mechanism for this method relies on parallel pairing of DNA strands, which is biologically unlikely given the conditions of the reaction and that there is no known enzyme that could facilitate this reaction. Additionally, even if such reaction could take place, the results shown in Figure 4 are inconsistent with the expected product and therefore do not support the conclusions of the article regarding the performance and validation of the reported approach.

All Authors agree with this retraction.

